# Prosthetic Rehabilitation of All-on-Six Implant-Supported Prosthesis: A Case Report

**DOI:** 10.7759/cureus.51946

**Published:** 2024-01-09

**Authors:** Dhanashree A Minase, Seema Sathe, Anjali Bhoyar, Aditee Apte, Ankita Pathak

**Affiliations:** 1 Prosthodontics, Sharad Pawar Dental College, Datta Meghe Institute Of Higher Education, Wardha, IND

**Keywords:** implants, fixed prosthesis, implant supported prosthesis, paulo maulo, full-mouth rehabilitation

## Abstract

This clinical report explores the effectiveness of dental implants for rehabilitating fully edentulous arches, with a focus on the all-on-six treatment approach. Implant-supported fixed restorations, particularly using six implants, are presented as an expected and cost-effective solution for the rapid repair of the edentulous patient, avoiding the need for bone grafting. This report details the successful rehabilitation of a patient's completely edentulous arches using the all-on-six concept, highlighting the meticulous planning and execution involved. It concludes that precise diagnostic and implant planning, along with thorough attention to all the features, is crucial for successful implant-supported fixed prostheses, with the all-on-six concept offering improved clinical and radiological outcomes for atrophied maxillae.

## Introduction

Implementing the widespread use of dental implants for the rehabilitation of fully edentulous arches has become relatively popular. Full arch rehabilitation can be accomplished with either a fixed or detachable overdenture prosthesis [1,2]. Implant-supported fixed restorations are an accepted treatment option for edentulous patients. Long-term clinical investigations have demonstrated that this form of repair can be effective for many years [3-5]. Six implants, according to Agliardi and colleagues, can be considered a predictable, cost- and time-effective solution for rapid repair of the edentulous jaw, avoiding bone grafting treatments [6]. The all-on-six treatment approach demonstrates the best biomechanical behaviour and is a viable option for moderate atrophic jaw rehabilitation [7].

## Case presentation

**Figure 1:**
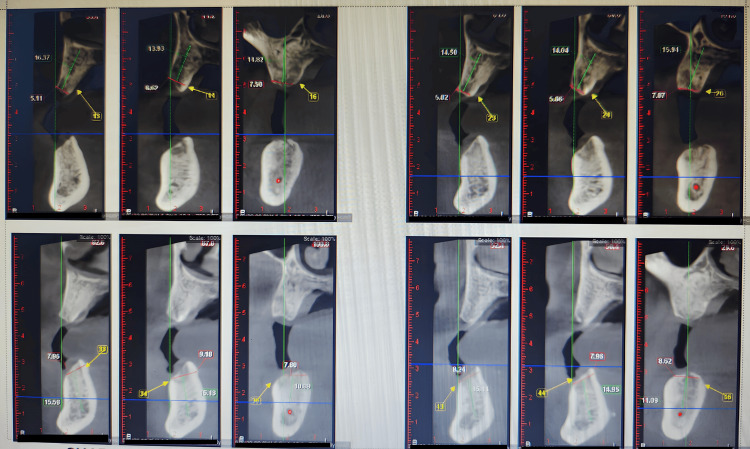
Cone beam computed tomography (CBCT)

A 53-year-old male patient reported complete edentulous maxillary and mandibular arches to the prosthodontics department. He didn't like the removable prosthesis due to poor retention and desired a fixed prosthesis. After a complete examination of the bone, which was D2 type in both the arches (porous cortical and coarse trabecular according to Misch classification), a treatment plan was decided to place six implants in the maxilla and six implants in the mandible. Implant planning was done after the cone beam computed tomography (CBCT) examination as shown in Figure 1.

**Figure 2:**
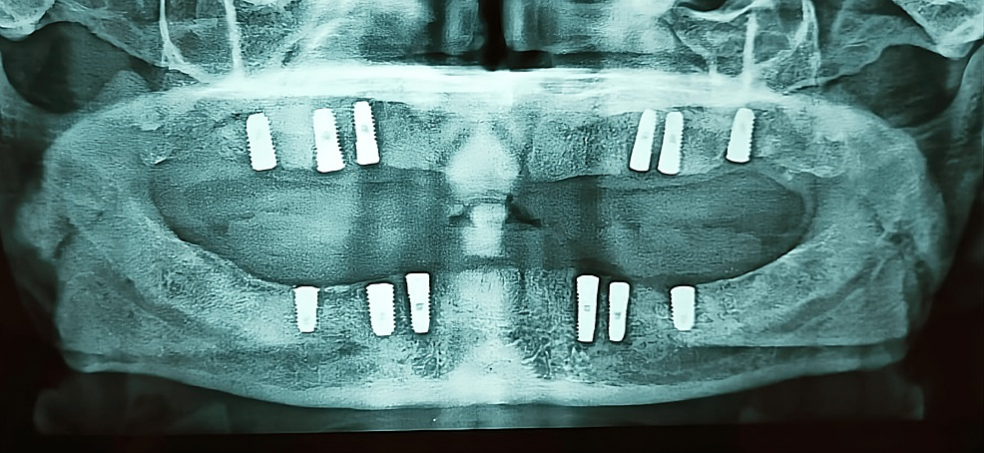
Radiograph showing six implants each in maxilla and mandibular arch

Six implants were placed in the 13, 14, 16, 23, 24, and 26 regions of size (in diameter and length) 4.0x13 mm, 4.5x11.5 mm, 5.0x10 mm, 4.0x13 mm, 4.5x11.5 mm, 5.0x10 mm in the maxilla and six implants were placed in 33, 34, 36, 43, 44, 46 regions of size 4.0x13 mm, 4.5x11.5 mm, 5.0x10 mm, 4.0x11.5 mm, 4.5x11.5 mm, 5.0x10 mm in the mandible depending upon the amount of bone present. The final prosthesis was given after three months of implant placement due to less implant stability at the time of insertion. The radiograph is shown in Figure 2.

**Figure 3:**
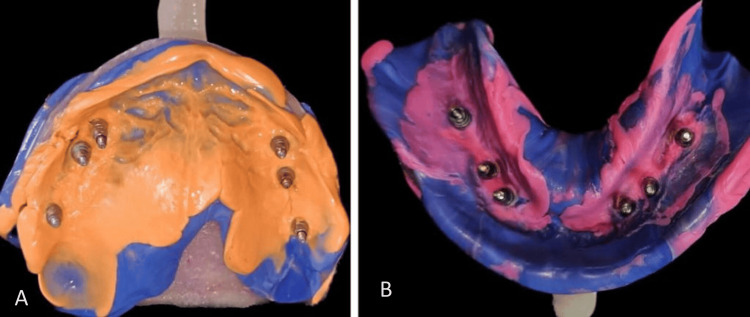
Open tray impression of (A) maxillary and (B) mandibular arches

An alginate impression was made for diagnostic models upon which a custom-made open tray was fabricated. Intraorally, open tray impression coping was attached to the implant after removing the healing caps. These copings were splinted intraorally together to give increased rigidity as well as possibly higher precision. The open tray impression was made using elastomeric material, excess material above the impression coping was removed, and unscrewing of the impression coping was done once the impression material was set (Figure 3).

**Figure 4:**
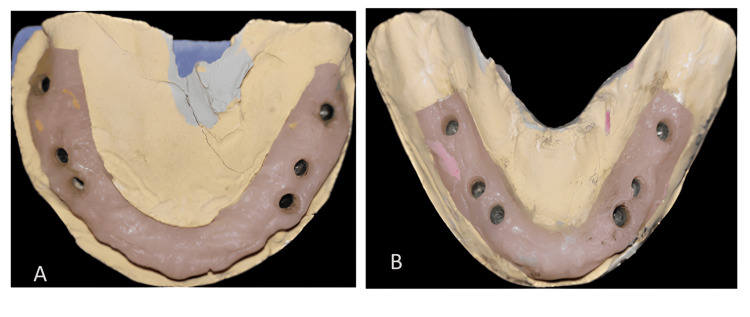
(A) Maxillary and (B) mandibular cast

The cast was poured after applying a gingival mask with type III dental stone (Figure 4).

**Figure 5:**
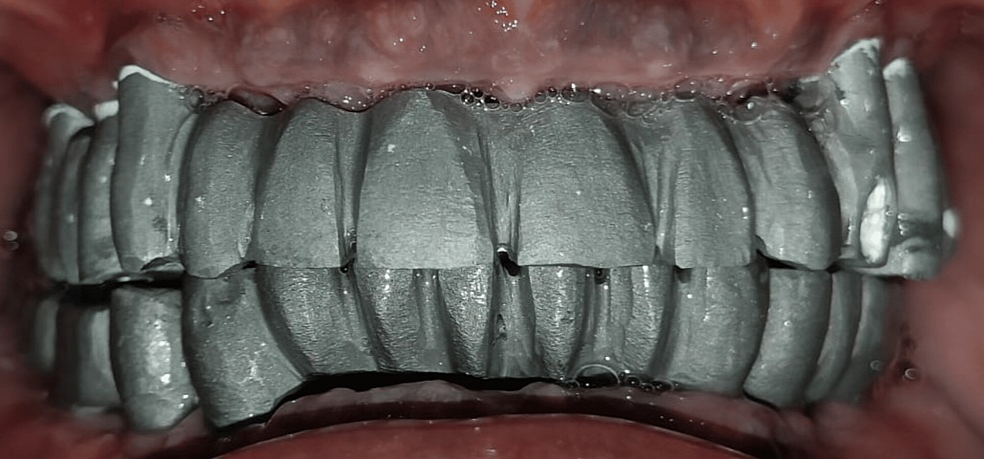
Metal trial performed intraorally

Jig verification was performed intraorally which was made on the diagnostic cast with the use of pattern resin. Jaw relation was recorded using record base and occlusal rims. The entire assembly was articulated and evaluation of interridge distance was done, which was 19 mm between maxillary and mandibular ridges. Implant-supported fixed prosthesis (FP-2) was planned accordingly. The metal trial was performed intraorally (Figure 5).

**Figure 6:**
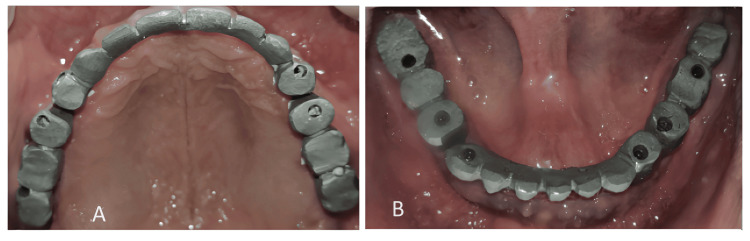
(A) Maxillary and (B) mandibular metal try-in with one anterior and two posterior bridge prosthesis

The fixed prosthesis was anterior from the first premolar to the first premolar region and two posterior from the second premolar to the second molar in both arches. The maxillary prosthesis was cement-retained and the mandibular prosthesis was screw retained for even distribution of the forces (Figure 6).

**Figure 7:**
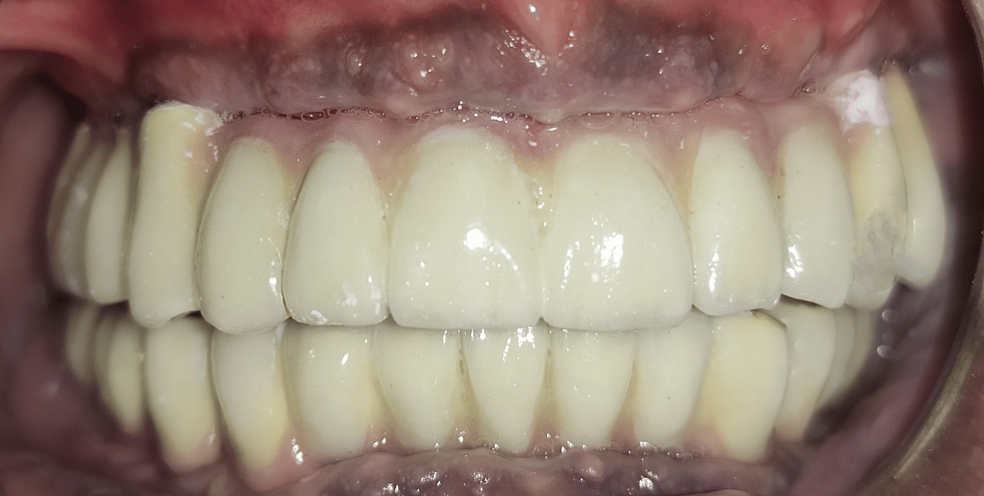
Final prosthesis insertion

The final prosthesis insertion was completed and a torque of 25 Ncm was given for screw-retained prosthesis. Occlusal adjustments were performed. The patient was scheduled for regular recall (Figure 7).

## Discussion

The all-on-four therapy allows for restoring a complete absence of teeth in the jaw with little bone volume, cheaper cost, short treatment intervals, improved quality of life, and lower patient morbidity [8,9]. Four implants are utilized to support fixed dentures in the restoration of totally edentulous jaws [10]. The all-on-four technique has an implant success rate of up to 99% and is predictable but prosthetic survivorship, on the other hand, is slightly lesser [11]. Porcelain crown failure, Prosthetic fracture, prosthetic screw loosening, abutment loosening, and the presence of a distal cantilever increase the likelihood of mechanical difficulties in the prostheses [12]. These disadvantages of all-on-four can be overcome by all-on-six prostheses. The all-on-six concept looks less stressful than the all-on-four [13]. The increased number of implants boosted prosthesis support and resulted in better stress distribution over a greater region [14].

The all-on-six treatment approach was created to increase the usage of accessible remaining bone in the jaw, allow function speedily, and avoid regeneration procedures that raise treatment costs [15]. As in all-on-six cases, no cantilever is present, the risk of biomechanical complications such as prosthetic screw loosening, and implant overload is reduced. Hassan and coworkers concluded that the all-on-six implant idea is superior to the all-on-four implant concept for atrophied maxilla because it was analogous with enhanced clinical and radiological parameters in their one-year study [16].

The implant placement was done by the prosthodontic plan. In this patient, a six-unit implant-supported anterior fixed dental prosthesis (FPD), and two three-unit posterior implants supported the FPDs. The advantages of segmenting the FPDs into three FPD units versus a one-piece prosthesis were lower maintenance expenses, lower cost of follow-up (in the event of loss of abutment, chipping, or porcelain fracture), and improved distribution of occlusal and lateral loads [17-19]. According to Park et al., implants can be successfully placed in elderly individuals and long-term prognosis is also good; he concluded that age is not the only factor for the success of implants [20].

As no cantilevers were placed in all-on-six prostheses, high survival rates are seen compared to all-on-four cases. Cantilever presence raises the likelihood of biomechanical complications such as prosthetic screw loosening and implant overload. As a result, additional implants are favourable for improving prosthetic support and decreasing the length of the cantilever [21]. In the study by Agliardi et al., all-on-four groups had significantly higher plaque than the all-on-six groups. This could be attributable to the hybrid of four fixed restoration's complex oral hygiene due to the presence of broad flanges of the prosthesis, which cause increased plaque formation [22]. Direct metal laser sintering, a computerized way for fabrication, has been employed in the current patient as a substitute to traditional traditional partial denture prostheses. It is novel and provides exact restorations, simplifies post-processing procedures, and is devoid of casting defects [23].

## Conclusions

As the stresses were decreased in the all-on-six planning than in the all-on-four design and the cantilever was also reduced, it is deemed favourable. Hence, all-on-six could be said to be a better treatment option in edentulous patients who desire fixed prostheses. This treatment is a good alternative for individuals with considerable tooth loss since it can significantly enhance their dental function, comfort, and aesthetics while enhancing their quality of life. The all-on-six implant system can provide a personalized and natural-looking smile that fits perfectly with the patient's facial features, increasing self-confidence and satisfaction with life. So, we can say that this treatment option can be considered as a future of denture prosthesis.
